# Optimization of ERK Activity Biosensors for both Ratiometric and Lifetime FRET Measurements

**DOI:** 10.3390/s140101140

**Published:** 2014-01-10

**Authors:** Pauline Vandame, Corentin Spriet, Franck Riquet, Dave Trinel, Katia Cailliau-Maggio, Jean-François Bodart

**Affiliations:** 1 Laboratoire de Régulation des Signaux de division, EA4479, Institut Fédératif de Recherche (IFR) 147, Site de Recherche Intégré en Cancérologie (SIRIC) ONCOLILLE, University Lille1, Villeneuve d'Ascq F-59655, France; E-Mails: pauline.vandame@ed.univ-lille1.fr (P.V.); franck.riquet@iri.univ-lille1.fr (F.R.); Katia.Maggio@univ-lille1.fr (K.C.-M.); 2 Interdisciplinary Research Institute, USR3078 CNRS, Université Lille Nord de France, 50 avenue de Halley, Villeneuve d'Ascq Cedex F-59558, France; E-Mails: corentin.spriet@iri.univ-lille1.fr (C.S.); dave.trinel@iri.univ-lille1.fr (D.T.)

**Keywords:** genetically-encoded biosensor, ERK, FRET

## Abstract

Among biosensors, genetically-encoded FRET-based biosensors are widely used to localize and measure enzymatic activities. Kinases activities are of particular interest as their spatiotemporal regulation has become crucial for the deep understanding of cell fate decisions. This is especially the case for ERK, whose activity is a key node in signal transduction pathways and can direct the cell into various processes. There is a constant need for better tools to analyze kinases *in vivo*, and to detect even the slightest variations of their activities. Here we report the optimization of the previous ERK activity reporters, EKAR and EKAREV. Those tools are constituted by two fluorophores adapted for FRET experiments, which are flanking a specific substrate of ERK, and a domain able to recognize and bind this substrate when phosphorylated. The latter phosphorylation allows a conformational change of the biosensor and thus a FRET signal. We improved those biosensors with modifications of: (i) fluorophores and (ii) linkers between substrate and binding domain, resulting in new versions that exhibit broader dynamic ranges upon EGF stimulation when FRET experiments are carried out by fluorescence lifetime and ratiometric measurements. Herein, we characterize those new biosensors and discuss their observed differences that depend on their fluorescence properties.

## 1. Introduction

How a cell can integrate numerous external signals to elicit a specific and adapted response with only a limited number of signaling effectors remains a puzzling question. These effectors are finely tuned and regulated by post-translational modifications, including phosphorylation, which in turn is able to control subcellular localization and activity. Intensity and duration of the phosphorylation depend on the equilibrium between the activities of kinases and phosphatases, as well as on the crosstalk and connectivity established between pathways. All together, these parameters ensure appropriate and specific cell decisions.

Among the signaling cascades of phosphorylated molecules, which are dedicated to conveying information and integrating extracellular signals, the Mitogen Activated Protein Kinase (MAPK) signaling network stands out [1,2]. Each MAPK signaling chain is a three tier cascade in which the upstream kinase (MEKK) phosphorylates and activates a second one (MEK), which in turn, phosphorylates and activates a third kinase (MAPK). The MAPK/ERK cascade successively enrolls MOS or Raf, MEK1/2, Extracellular Regulated Kinase (ERK) 1/2 that activates by phosphorylation a plethora of substrates within the cytoplasm and the nucleus, as effectors involved in the physiological response and phenotypical outcome. These substrates include transcription factors, kinases and phosphatases, signaling proteins, cytoskeletal proteins, proteinases and apoptotic proteins [3]. These substrates can be expressed in a cell specific type manner, but their activation also relies on threshold effects, therefore, the duration and amplitude of ERK activity level are crucial for signal integration. Depending upon the presence of feedbacks, the cascade may display different kinds of temporal responses affecting the resulting phenotypes. Indeed, distinct profiles of ERK activation in response to different growth factors were revealed in PC12 cells: EGF stimulation leads to a transient ERK activation, whereas NGF induces a sustained activation [4]. Network rewiring can also drive an abrupt ERK response in models like *Xenopus* oocytes, where feedback loops and feedforward loops elaborate physical properties like ultrasensitivity, bistability and irreversibility [5–7]. Spatial regulation also participates in proper signal propagation. Several scaffolding proteins that bind the components of the MAPKs cascade have been identified. They participate in anchoring the kinases and can promote their activation in different cell compartments. For example Sef1 localizes MEK on the Golgi membrane and KSR assembles the three members at the plasma membrane following the pathway stimulation [8].

During the past two decades, new tools have emerged concomitantly to the discovery of GFP. Among these tools, genetically encoded enzyme activity reporters based on Förster Resonance Energy Transfer (FRET) imaging have become more and more attractive due to their ability to sense and report the level of several analytes such as second messengers, ions or protein activity in living cells or tissues [9]. FRET is a non-radiative energy transfer between a pair of fluorophores. The energy transfer depends on the spectral overlap between the emission and excitation spectra, and the relative orientation of the so-called donor and acceptor. This energy transfer can only occur if donor and acceptor are in close vicinity. Genetically encoded enzyme activity reporters based on FRET are biosensors, since the latter term include systems composed of at least two parts: one part that specifically recognized an analyte, and a second part which “transduces” and conveys the signal from the recognition site toward an adapted instrument collecting a measurable signal.

Kinase activity biosensors are constituted by two fluorophores adapted for FRET experiments. Those fluorophores are flanking a specific peptide substrate of ERK and a domain recognizing and binding this peptide substrate when phosphorylated. The latter recognition allows a conformational change of the biosensor and thus a FRET signal.

Upon FRET several properties of light are modified and can be measured. The emission intensity of the donor fluorophore decreases, while emission intensity of the acceptor increases. In this way FRET can thus be evaluated by dividing the YFP signal by the CFP signal. An increase of this YFP/CFP ratio thus corresponds to an increase of kinase activity and *vice versa* [10]. The lifetime of the donor fluorophore is also affected by a FRET event and decreases upon kinase activity.

The purpose of optimizing such tools directly relies on kinase activity behavior. In fact, in some biological processes, kinase activity changes are too low to be detected with the existing biosensors. This depends directly on an intrinsic property of the biosensor called the dynamic range, which corresponds to the maximal difference of “FRET value” between two conditions: when there is no kinase activity and thus no phosphorylation of the biosensor and when kinases are fully activated and thus biosensors are folded and give a strong signal. Dynamic range corresponds thus to the ability of the biosensor to give a measurable FRET signal even when kinase activity is low.

Regarding MAPK/ERK signaling pathway, the first FRET-biosensor designed was named Miu2 (for MAPK Indicator Unit ERK2) [11]. This biosensor used the conformational change of ERK occurring upon the binding of its activator MEK. Miu2 was made up with a FRET pair (CFP and YFP) flanking the *Xenopus* ERK2 sequence, from which they were spaced by short linkers of two and three aminoacids. When the MAPK/ERK pathway cascade is recruited by an external signal, activated MEK binds to endogenous ERK as well as the ERK enclosed within the sensor in order to achieve phosphorylation. This binding lead to a modification of ERK conformation within Miu2, bringing closer the two fluorophores and therefore noticeably increasing the FRET signal. However, Miu2 expression acts as an overexpression of ERK2, which is a disadvantage because ERK2 overexpression disturbs cellular processes like in the case of stimulation of proliferation in human hepatocellular carcinoma study [12]. Moreover if Miu2 reflects MEK activity and MEK-ERK interaction it does not reflect *per se* ERK activity. This last issue was resolved in 2007 with the engineering of Erkus [13], whose principle was quite different from Miu2, since the latter is based on the interaction between a phosphorylated substrate with a domain recognizing this phosphorylation. Erkus was built using the same fluorophore pair as Miu2, but these fluorophores were flanking a short amino acid sequence, corresponding to a specific target of ERK (threonine 669 within the Epidermal Growth Factor Receptor), followed by the FHA2 (Forkhead-Associated 2) domain, which binds to phosphorylated threonine residues. Moreover, an ERK docking motif was introduced at the end of the acceptor fluorophore sequence, to increase the affinity of ERK to the biosensor. Upon ERK activation, the substrate is phosphorylated and then recognized by the phospho-aminoacid binding domain (PAABD), leading to a conformational change that allows FRET phenomenon between the fluorophores. Such a process remains reversible upon the action of specific phosphatases or inhibition of kinase activity [13].

In 2008, another ERK biosensor named Extracellular-regulated Kinase Activity Reporter (EKAR) was published by Harvey and collaborators [14], taking advantages of the elements of Erkus but adapting each component. EKAR FRET pair was composed of a CFP variant (mCerulean) and of a YFP variant (mVenus), since constant progress in FPs engineering allows for an improved FRET efficiency by changing the fluorophore pair of a biosensor. Alternatively a biosensor was made up using another couple, eGFP and mRFP. The flanked sequence by the fluorophores includes in that case: (i) a partial sequence of the Cdc25C substrate containing the threonine 48 residue [2,15]; (ii) a WW domain, which replaced the FHA2 domain and also recognizes phosphorylated threonine residues but with an increased affinity for the proline-rich sequence; (iii) a linker constituted of 72 glycine residues was inserted between these two parts in order to improve the flexibility and favor the folding of the biosensor. The resulting EKAR revealed higher FRET variation than Erkus and Miu2. Finally in 2011, Komatsu and collaborators developed an optimized backbone for the generation of activity reporters [16]. The major characteristic of this backbone was the development of an improved linker whose length was optimized in order to favor the energy transfer between the two FRET pairs (e.g., ECFP/YPet or Turquoise-GL/YPet). Thus, development of ERK biosensors reflects the constant need for optimized tools to analyze kinase behaviors during cellular processes. It relies notably on optimizing their dynamic range. It corresponds to the maximal interval of variation that can be obtained by measuring the FRET level when there is no kinase activity and upon a strong stimulation of kinase activity. Thus, an increased dynamic range that allows detecting finer variations will increase our understanding of protein dynamics and cell fate decisions.

The dynamic range of the preexisting MAPK biosensors to be optimized is directly associated with FRET efficiency [9], which, in the case of a biosensor, relies: (i) from a structural point of view, on the folding of the biosensors when phosphorylated/dephosphorylated (*i.e.*, orientation factor, distance between fluorophores) and (ii) from a photophysical point of view, on the fluorophores' properties (*i.e.*, quantum yield, overlap integral). Two fluorescence properties, modified by FRET, are measured: fluorescence emission is monitored by ratiometric measurements while fluorescence lifetime is measured in Fluorescence Lifetime Imaging Microscopy-FRET (FLiM-FRET). Here we report new ERK biosensors, namely EKAR-Cep-CpV, EKAR-TVV and EKAREV-TVV, modified from the two last generation of biosensors EKAR and EKAREV [14,16]. Those new probes have been tested and compared with two different FRET techniques, fluorescence intensity and lifetime based measurements, in order to provide the most versatile ERK activity biosensor.

## 2. Experimental Section

### 2.1. DNA Constructs

Three different constructs were produced based on EKAR and EAKREV sequences [14,16], taking benefit of recent tandem fluorophores [17,18], and named accordingly to their structural elements. EKAR-Cer/CpV was generated by subcloning the coding sequence of the molecular recognition element (eg: docking domain followed by phospho-aminoacid binding domain, 72-Gly linker and substrate) of prK5 cEKAR [14], inserted between the two fluorophore sequences of the pcDNA3 AKAR4 [18]. First the AKAR4 coding sequence was sub-cloned into a pCS2 vector using BamHI/EcoRI. The BamHI unique restriction site of pcDNA3 was deleted by Klenow fill-in. Second, a BamHI restriction site was reintroduced at the 3′ extremity of the Cerulean coding sequence by site-directed mutagenesis using the 5′-CTGTTCTTGAGAAAACTTATGGATCCGCTTGTACAGCTCGTCCATG-3′ primer. The obtained plasmid contains thus two unique restriction sites BamHI and SacI flanking the whole sequence between the two fluorophores of AKAR4. The molecular recognition element of *prK5 cEKAR* was amplified by PCR using the sense-oligonucleotide 5′-AGCGGATCCATATGGCGGACGAGGAGAAGC-3′ and the antisense-nucleotide 5′-CATGAGCTCGATATCCCGGGCCCGCGG-3′ containing, respectively, one BamHI and one SacI restriction site. The resulting fragment was then subcloned into the pCS2 AKAR4 vector using BamHI/SacI enzymes.

### 2.2. EKAR-TVV

This was generated using the same kind of strategy: sub-cloning the sequence of the molecular recognition element of EKAR [14] between the FRET pair sequence of another biosensor named ^T^EPAC^VV^ [17]. The whole sequence between the fluorophores of the pCDNA3 ^T^EPAC^VV^ is surrounded by two unique restriction enzymes EcoRV and NheI. Because those restriction sites were already present in the coding sequence of EKAR, we proceed to a multisites-directed mutagenesis to delete those sites by introducing two silent mutations in EKAR with two primers: 5′-GGAGGAGGAGGAGCCAGCGGCGG AGGTGG-3′ and 5′-GCGGGCCCGGGACATCATGGTGAGCAAGG-3′. The molecular recognition element sequence of prK5 cEKAR was then amplified by PCR using the sense-oligonucleotide 5′-CGAT ATCTCCGGATCCATATGGCGGACGAG-3′ and the antisense-nucleotide 5′-CGCTAGCGATGTCC CGGGCCCGCGGG-3′ containing respectively EcoRV and NheI. The resulting fragment was then sub-cloned into the pCDNA3 ^T^EPAC^VV^ vector.

### 2.3. EKAREV-TVV

As the FRET pair used in the second construction gives us good results, we tried to optimize it with an improved linker named EV-linker contained in another MAPK biosensor EKAREV [16]. The major difference between the two first generations of MAPK biosensors [14,16] consists in a linker variation between the MAPK subtrate and the WW domain which serves as phosphor amino-acid binding domain. To obtain a sensor containing both the FRET pair mTurquoise and CpVenus172-Venus and the EV-linker we amplified the whole sequence surrounded by the fluorophores of EKAR-EV by PCR using the following primers 5′-CGATATCTCGAGATGGCGGACGAGGAGAAG-3′ and 5′-CGCTAGCGCGG CCGCCCGGAAATTG-3′ containing EcoRV and NheI restriction sites, respectively. The PCR fragment obtained was once again sub-cloned between the fluorophores of the pCDNA3 ^T^EPAC^VV^. The same procedure was used to obtain the negative control sensor. We amplified the mutated (Threonine to Alanine within the substrate) EKAREV and sub-cloned this fragment into the pCDNA3 ^T^EPAC^VV^.

### 2.4. Cell Culture, Transfections and Chemicals

Cells were cultured in Dulbecco's modified Eagle's medium supplemented with 10% foetal calf serum and antibiotics (100 units/mL of penicillin and 100 μg/mL of streptomycin) at 37 °C with 5% CO2. Cells were seeded on 32 mm coverslips in six-well plates and grown to reach 50% of confluency. Cells were then transfected with 1 μg of DNA per well using FuGENE HD transfection reagent (Roche, Mannheim, Germany). Twenty four hours later, cells were starved in DMEM with 0.5% FCS overnight. Prior to images acquisition, DMEM was removed and cells were placed in L-15 buffered medium. Epidermal Growth Factor (R&D Systems, Minneapolis, MN, USA) was resuspended in PBS and used at 100 ng/mL final concentration. MEK inhibitor U0126 dihydrocholride (Sigma, St. Louis, MO, USA) was resuspended in DMSO and used at the final concentration of 50 μM.

### 2.5. Ratiometric Measurements

Cells on coverslips were placed on a thermostatted (37 °C) inverted Leica AF6000 videomicroscope (Leica Microsystems, Wetzlar, Germany) with 63×, 1.3 numerical aperture (NA) glycerin-immersion objective. Fluorescence excitation was performed with a 427 ± 10 nm bandpass filter through a double band dichroic mirror (440/520 nm). To avoid displacements between donor and acceptor's fluorescence measurements, we used fast detection filter wheel (respectively with 472 ± 30 nm and 542 ± 27 nm bandpass filters). Images were acquired every two minutes. After 6 min, drugs were added to the cell medium by gentle pipetting.

YFP/CFP ratio for cell by cell data analysis was realized with the ImageJ software as described in [10]. Curves were obtained by subtracting the average of the baseline values (time points 0 to 6 min) from each time point. Histograms were calculated by subtracting the same average of baseline values from the average of plateau values after EGF stimulation (points at time 12 to 20 min). In both cases, average and standard deviation are calculated and represented. *p*-values were obtained using Student t-test.

### 2.6. FLiM Measurements

Frequency domain FLiM experiments were performed using the LIFA system (Lambert Instruments, Roden, The Netherlands) assembled on a wide field Nikon Eclipse TE2000 inverted microscope (Nikon, Amsterdam, The Netherlands) in a 37 °C thermostatted chamber and with a 60×, 1.4 numerical aperture (NA) oil immersion objective and appropriate CFP filter cube (excitation 426/446 nm; dichroic mirror 455 nm; emission 460/500 nm). The modulation frequency was set to 40 MHz and MCP to 800 V. We used a solution of 7-(diethylamino)coumarin-3-carboxylic acid (Sigma) diluted in methanol as lifetime reference (4 ns). 36 phases shifted intensity images were recorded every two minutes, pre-diluted EGF and U0126, according to the experimental designs, were manually added into the culture medium. Lifetime values were extracted with LiFliM (Lambert Instruments). For each cell, lifetime variation was obtained by subtracting the average of the baseline values (time points 0 to 6 min) from each time point. Histograms represent the variation before (time points 0 to 6 min) and after (10–20 min) EGF induction. In both cases the average and standard deviation is calculated and represented. *p*-values were obtained using Student t-test.

## 3. Results and Discussion

**Figure 1.**
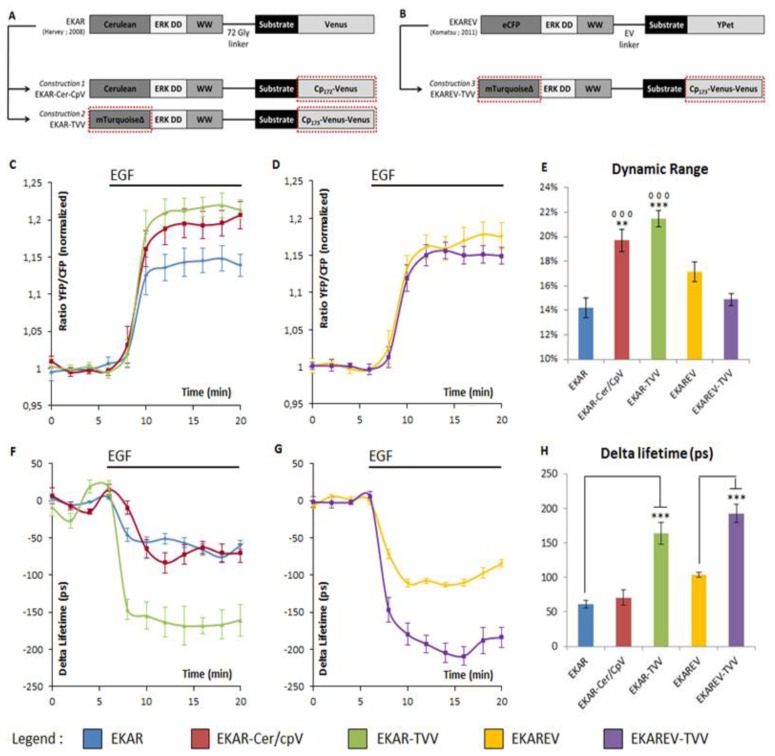
Design and properties of ERK biosensors EKAR-Cer/CpV, EKAR-TVV and EKAREV-TVV.

The choice of the FRET pairs was guided by previous works on other FRET-based biosensors, one reporting PKA activity [18] and a second one probing the intracellular cAMP level [17]. Concerning the PKA activity biosensors, the optimization from A Kinase Activity Reporter 3 (AKAR3) to AKAR4 consisted in a swapping of the donor fluorophores eCFP for the Cerulean FP in combination with a circularly permutated Venus (in position 172) [18,19]. This change improved the dynamic range of the biosensor from 38% to 58% in ratio experiments [18]. Therefore, we used the same kind of procedure on EKAR and exchanged the Venus FP with its variant Cp172 Venus, to obtain a first construction named *EKAR-Cer-CpV*, including the Cerulean/cpVenus FRET pair (Figure 1A). ^T^EPAC^VV^ is a cAMP level FRET biosensor that exhibits a remarkable dynamic range. This probe allows one to observe variations up to 700 picoseconds in lifetime experiments and 100% ratio variation upon the stimulation of cAMP production [17]. Those performances drew our attention on the FRET pair used in this sensor: A combination of mTurquoise and a dimer cpVenus-Venus. The donor fluorophore is characterized by a phase lifetime of 3.7 ns (instead of 2.3 for the Cerulean) [20,21], which improves FLIM measures, while the use of an acceptors dimer also improves ratiometric experiments. We thus generated a second construction based on this pair and named it *EKAR-TVV* (Figure 1A).

As the ratiometric techniques is widely used for FRET-based biosensor measurements compared to FLiM experiments, we choose to first test *EKAR-Cer-CpV* and *EKAR-TVV* with this technique. Cells were starved in order to inhibit ERK activity and then stimulated with Epithelial Growth Factor (EGF). The difference between the basal ratio values corresponding to starved cells and the ratio values following EGF stimulation corresponded to the dynamic range of the biosensors. In order to compare the results among themselves, each value obtained over time for every single cell, was normalized on the average of baseline values (Figure 1C,D). When compared to EKAR, our two constructions exhibited an increased dynamic range as shown by the normalized YFP/CFP ratios after EGF stimulation (Figure 1C). To highlight those changes the average of baseline values was subtracted to the average of the values upon EGF stimulation and was depicted in Figure 1E. EKAR-TVV in particular, revealed a 21.5% FRET increase upon EGF stimulation, when EKAR-Cer-CpV and EKAR reached respectively 19.7% and 14.2% (Figure 1C,E).

Those probes were then tested in frequency domain FLiM. As previously described, cells were starved and then stimulated with EGF, and images were acquired every minute. Curves thus represented the lifetime variation to the average of baseline values (Figure 1F,G). The difference between baseline values and EGF values was also represented for each biosensor (Figure 1H). EKAR-TVV's dynamic range was higher than EKAR's (160 ps and 60 ps respectively) whereas there was no significant improvement with EKAR-Cer-CpV.

To summarize, our ERK sensor EKAR-TVV, based on the molecular recognition element of EKAR coupled to a FRET pair composed of mTurquoise and a dimer CpVenus-Venus FPs, exhibited an increase dynamic range in both techniques contrary to EKAR-Cer-CpV, which was improved for ratio experiments but did not show any difference with the original sensor when tested in FLiM.

The previously reported MAPK sensor EKAREV [16] was also an improved version of EKAR notably due to the modification of a linker within the probe. We thus choose to combine this modification with the most efficient FRET pair to further increase the biosensors dynamic range (Figure 1B). This third construction, named EKAREV-TVV, did not show improvement compared to EKAR or EKAREV using ratiometric techniques (Figure 1D,E). Conversely, it strongly improved the dynamic ranges in lifetime-based experiments compared to EKAREV (192 ps and 100 ps respectively). Moreover EKAREV-TVV overrided EKAR-TVV performance (160 ps), making it our best construction for lifetime experiments (Figure 1G,H).

Figure 1A,B illustrates the design of new ERK biosensors. **ERK DD** corresponds to an ERK docking domain (amino acid sequence: FQFP). **WW** is a phospho-aminoacid binding domain. The substrate corresponds to a part of the Cdc25C sequence, coding for the amino acid sequence PDVRTPVGK specifically recognized and phosphorylated by ERK on the Threonine 48 residue. Dotted red boxes highlight the changes bring to the original constructions. EKAR-Cer/CpV and EKAR-TVV derive respectively from EKAR [14] (A) and EKAREV-TVV derives from EKAREV [16] (B).

Figure 1C,D shows the YFP/CFP ratio variations upon EGF stimulation in starved HeLa cells. EKAR-Cer/CpV (n = 43 cells) and EKAR-TVV (n = 23 cells) are compared to EKAR (n = 24 cells) (C). EKAREV-TVV (n = 22 cells) is compared to EKAREV (n = 28 cells) (D). The histogram summarizes the percentage of FRET variations upon EGF stimulation (E). Two symbols represent a *p* value ≤0.01 and three symbols represent a *p* value ≤0.001 when compared to EKAR (circles) or EKAREV (stars).

Figure 1F,G shows the fluorescence lifetime variations upon EGF stimulation in starved HeLa cells. EKAR-Cer/CpV (n = 6 cells) and EKAR-TVV (n = 8 cells) are compared to EKAR (n = 7 cells) (F). EKAREV-TVV (n = 5 cells) is compared to EKAREV (n = 4 cells) (G). The histogram sums up the variation of the phase lifetime upon EGF stimulation (H). Three stars represent a *p* value ≤0.001 when compared to EKAR or EKAREV.The YFP/CFP ratio was normalized on the average of the baseline values (time points 0 to 6 min) for each cell (C, D). Lifetime variation was obtained by subtracting the average of the baseline values (time points 0 to 6 min) from each time point for each cell (F, G). For each time point either the average YFP/CFP ratio or the average delta lifetime of n cells and the standard deviation were calculated and represented. Histograms (E, H) were calculated by subtracting the same averages of baseline values from the average of plateau values after EGF stimulation (points at time 12 to 20 min). *p*-Values were obtained using Student t-test.

**Figure 2.**
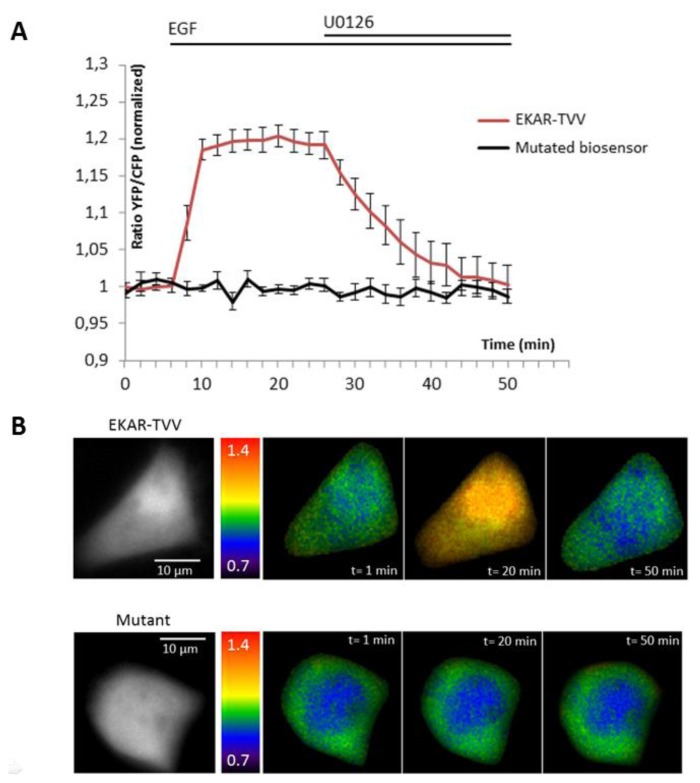
Reversibility control of EKAR-TVV in ratio experiments. Curves show the YFP/CFP ratio variations upon EGF stimulation and then inhibition of MAPK/ERK pathway with U0126 in starved HeLa cells expressing EKAR-TVV (n = 16 cells) or a mutated biosensor (n = 25 cells) (**A**). A representative cell illustrates the response of EKAR-TVV (upper panel) and the mutant (lower panel). Cells are represented before and after EGF stimulation and then after U0126 inhibition, in pseudo-color scale that corresponds to ratio values. Left panel represents CFP intensity in grey level (**B**).

**Figure 3.**
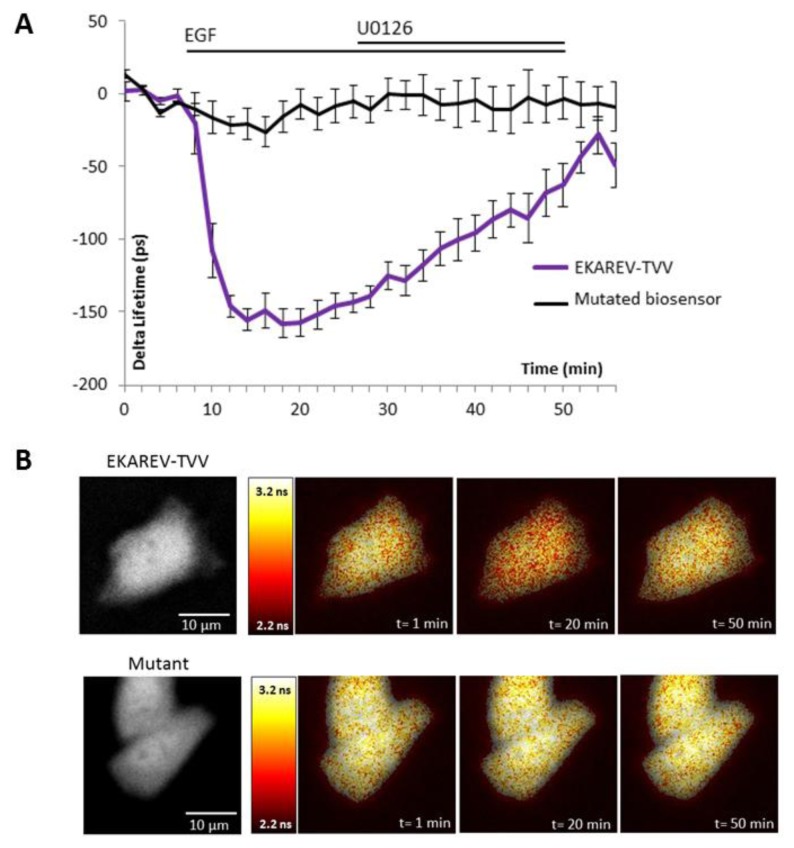
Reversibility control of EKAREV-TVV in FLiM experiments. Curves show the lifetime variations upon EGF stimulation and then inhibition of MAPK/ERK pathway with U0126 in starved HeLa cells expressing EKAREV-TVV (n = 8 cells) or a mutated biosensor (n = 6 cells) (**A**). A representative cell illustrates the response of EKAREV-TVV (upper panel) and the mutant (lower panel). Cells are represented during baseline, EGF stimulation and U0126 inhibition, in pseudo-color scale that corresponds to fluorescence lifetime values. Left panel represents CFP intensity in grey level (**B**).

The ability of the biosensor to be dephosphorylated and return to its original conformation, when kinase activity decreases or stops, is a critical parameter. Since many kinases are regulated by negative feedback loops or more specifically if we take the sustained/transient activation of MAPK, a non-reversible probe would lead to wrong interpretation of physiological events, because we will be only able to detect the first activation step. To reverse the signal, the biosensor in its folded conformation has to allow the phosphatases to access to the phosphorylated site within the probe. We thus tested our best construction for each technique upon inhibition of the pathway by a chemical inhibitor of MEK, U0126 (Figures 2 and 3). Moreover, the relevance of kinase activity measurements needed to be controlled using a negative control biosensor. We obtained this probe by site-directed mutagenesis that replaced the phosphorylable amino acid within the substrate of the biosensor by a non-phosphorylable residue (e.g.,: threonine replaced by an alanine). We thus used a mutated biosensor to provide the evidence that FRET signal (increase or decrease) were not artifacts but corresponded to conformational changes of the probe (Figures 2 and 3).

EKAR-TVV revealed the highest dynamic range in FRET ratio study. Following an EGF stimulation cells expressing this probe, cells were exposed to the MEK inhibitor U0126. The CFP/YFP ratio returned to a basal level when MAPK were inhibited, which demonstrated that EKAR-TVV sensing was reversible. The variations were compared to the mutated biosensor (Figure 2A). A representative cell expressing either EKAR-TVV or the mutated biosensor before and after drugs addition allowed us to localize activity variations (Figure 2B). We did the same experiment in FLiM with the sensor that exhibited in our hands the higher dynamical range for this technique: EKAREV-TVV. As expected following an exposure to U0126, the fluorescence lifetime increased to reach a basal level corresponding to ERK activity when cells were starved (Figure 3A). As previously we used a mutated biosensor as control and plotted the lifetime variations for each construction on a representative cell (Figure 3B).

## 4. Conclusions

Increasing biosensor sensitivity may rely on different strategies optimizing the combination of sensing domains (substrate and PAABD), linkers and fluorophores. Each optimization process for a biosensor is challenging because the global dimension and topology of each of them is different, and thus the structural requirements of each biosensor are unique. In some cases, a systematic approach, building all the combinations of the sensors key elements might be carried out [22]. In other cases, one can anticipate the optimal conditions and thus narrow the elements to optimize. Our approach consisted in modifying the best available ERK activity biosensors, EKAR and EKAREV. While their sensing component consists of a compact domain, and thus presents low structural requirements [22] and while the linkers were previously optimized, one can predict that further optimization will rely on the chosen donor/acceptor FRET pairs. Since monitoring FRET-based biosensors relies mainly on fluorescence lifetime and intensity, we needed donor fluorophores with high fluorescence lifetime, and acceptors allowing strong quenching when in close vicinity.

Intensity and lifetime are two different properties of fluorescence. Intrinsic properties of fluorophores will therefore increase the dynamic range, *i.e.*, donor fluorescence lifetime for FLIM FRET experiments or both fluorophores brightness for ratiometric measurements. Thus, these properties can be used for a stepwise procedure of optimization of preexisting biosensors [14,16], in contrast to random approaches, which are time-consuming [22]. However, one should not discard the impact of other properties. One of the ideas that can be discussed relates to the fact the FRET biosensors may also be altered by quenching-related mechanism. If one considers the impact of conformational change on quenching, several cases may emerge: (i) quenching of donor—the dynamic range will increase in lifetime and ratiometric measurements; (ii) quenching of acceptor—the dynamic range of ratiometric measurements will be reduced whereas the lifetime measurements will not be impacted; (iii) quenching of both acceptor and donor—the signal to noise ratio will be reduced and therefore exert a strong negative impact on ratiometric measurements while the dynamic range of the lifetime measurements will be increased. That could explain why, surprisingly, when the expected-best FRET pair is combined to the expected-best linker, we only obtained a poor sensor for ratio experiments compared to FLiM.

Several requirements necessary to achieve the best dynamic range are independent of the measured properties of light, like the relative orientation of fluorophores. Due to the flexible nature of EKAR, it is hard to predict this orientation in a structural point of view. Then, while we can narrow the combination of what we identified as the best FRET pairs (CeruleanFP and mTurquoise as donors and Cp-Venus and Cp-Venus-Venus as acceptors [17,18]) and the best linkers (72 Gly and EV linker [14,16]), few empirical tests remained mandatory in the final optimization of such tools. We thus first tested the most promising FRET pairs and identified the mTurquoise/CpVenus-Venus (EKAR-TVV constructs) to give the higher dynamic range whatever the techniques. Then, we tested both available linkers in both lifetime and intensity based experiments. Our EKAR-TVV construction shows an increased dynamic range compared to the previous ERK biosensors EKAR and EKAREV in both ratiometric and lifetime experiments. On the other hand EKAREV-TVV is the best sensor for FLiM experiment, with almost 200 picoseconds of difference between inactive and active states. We were thus able to provide new optimized constructs depending on the measurement techniques. These biosensors will be a further milestone for a better understanding of ERK dynamics.

## Abbreviation List

72 gly: 72-glycine linker
AKAR3: A Kinase Activity Reporter 3
AKAR4: A Kinase Activity Reporter 4
cAMP: cyclic Adenosine Mono Phosphate
CFP: Cyan Fluorescent Protein
CpVenus172: Circularly permutated Venus in position 172
eCFP: Enhanced Cyan Fluorescent Protein
EGF: Epidermal Growth Factor
eGFP: enhanced Green Fluorescent Protein
EKAR: Extracellular signal-regulated Kinase Activity Reporter
EKAR-Cer-CpV: EKAR-Cerulean-Circularly permutated Venus
EKAREV: Extracellular signal-regulated Kinase Activity Reporter Enhanced Visualization
EKAREV-TVV: EKAREV-Turquoise-CpVenus-Venus
EKAR-TVV: EKAR-Turquoise-CpVenus-Venus
FHA2: Forkhead-Associated 2
FLiM: Fluorescence Lifetime Imaging Microscopy
FP: Fluorescent Protein
FRET: Förster Resonance Energy Transfer
GFP: Green Fluorescent Protein,
KSR: Kinase Suppressor of Ras
MAPK/ERK: Mitogen Activated Protein Kinase/Extracellular signal-regulated kinase
MAPK: Mitogen Activated Protein Kinase
MEK: MAPK/ERK protein Kinase
Miu2: MAPK Indicator Unit ERK2
NGF: Nerve Growth Factor
PAABD: PhosphoAminoAcid Binding Domain
PKA: Protein Kinase A
TEPACVV: Turquoise Exchange Protein directly Activated by cAMP CpVenus-Venus
WW domain: Tryptophan (W) repeated domain
YFP: Yellow Fluorescent Protein
